# Brief Report: Using the Internet to Identify Persons with Cognitive Impairment for Participation in Clinical Trials

**DOI:** 10.3390/brainsci7040036

**Published:** 2017-04-05

**Authors:** Lindsay F. Morra, Jason Brandt

**Affiliations:** The Johns Hopkins University School of Medicine, Psychiatry and Behavioral Sciences, Division of Medical Psychology, The Johns Hopkins Hospital, 600 North Wolfe Street, Meyer 218, Baltimore, MD 21287-7218, USA; jbrandt@jhmi.edu

**Keywords:** clinical trials, MCI, risk, dementia, Alzheimer’s, cognitive impairment, assessment

## Abstract

Identifying, recruiting, and enrolling persons in clinical trials of dementia treatments is extremely difficult. One approach to first-wave screening of potential participants is the use of online assessment tools. Initial studies using the Dementia Risk Assessment (DRA)—which includes a previously validated recognition memory test—support the use of this self-administered assessment to identify individuals with “suspected MCI” or “suspected dementia.” In this study, we identified between 71 and 622 persons with suspected dementia and between 128 and 1653 persons with suspected mild cognitive impairment (depending on specific criteria) over the course of 22 months. Assessment tools that can inexpensively and easily identify individuals with higher than average risk for cognitive impairment can facilitate recruitment for large-scale clinical trials for dementia treatments.

## 1. Introduction

Identifying, recruiting, and enrolling persons with varying degrees of cognitive impairment in clinical trials of dementia treatments is arduous, time-consuming, and very expensive [1,2,3]. This is especially true in multicenter and multinational studies that have become popular in recent years. Several screening methods have been developed and employed. These are typically comprised of informant questionnaires or cognitive tests, and are often administered in-person [4,5] or over the telephone [5,6,7,8,9,10,11,12].

One option that has become more feasible is the use of instruments on Internet websites for initial identification of potential participants [13,14]. Brandt and colleagues [15,16] have developed a web-based Dementia Risk Assessment (DRA) that obtains relevant medical history, assesses dementia risk factors, and performs a brief cognitive screen, all within 10 min. The results have been validated against other assessment instruments, as well as clinical diagnosis based on multiple diagnostic procedures in specialty dementia clinics [16,17]. We report here on the feasibility of using the DRA to identify persons with (a) suspected mild cognitive impairment (MCI) and (b) suspected dementia, who would then receive telephone or in-person screening for trial eligibility.

## 2. Materials and Methods

Version 2.0 of the Dementia Risk Assessment was launched on a free Internet website on 4 November 2014. This study has been granted ethical approval by the Johns Hopkins Medicine Institutional Review Board (#NA_00026550/CR00013493). Participants completed yes/no questions related to their medical, neurological, and psychological histories and health habits. They also took a brief verbal memory test involving the binding of six objects with incongruent attributes (e.g., “gray strawberry” and “blue sun”). After two to three minutes of additional questions, participants were administered a yes/no recognition memory test (RMT). Scores on the recognition memory test are calculated as the hit rate minus the false-positive rate, and range from +1.0 (perfect detection of target items and rejection of distracter items) to −1.0 (saying “no” to all targets and “yes” to all distracters).

Within 22 months of its launch, with very little publicity and no explicit direction of traffic to our website, 9409 people had completed the self-report version of the DRA (a proxy-report version is also available; fewer people have completed it, and it will not be discussed further in this report). The totally anonymous dataset was downloaded on 11 September 2016.

## 3. Results

Of the 9409 self-reports, 5769 were from persons between the ages of 50 and 100, and were presumed to be valid. Reasons for eliminating data as probably invalid were scores far out of range (e.g., height of 4′ tall, age 120 years) or extremely unlikely patterns (e.g., endorsing every disease but performing without error on the RMT).

**Figure 1:**
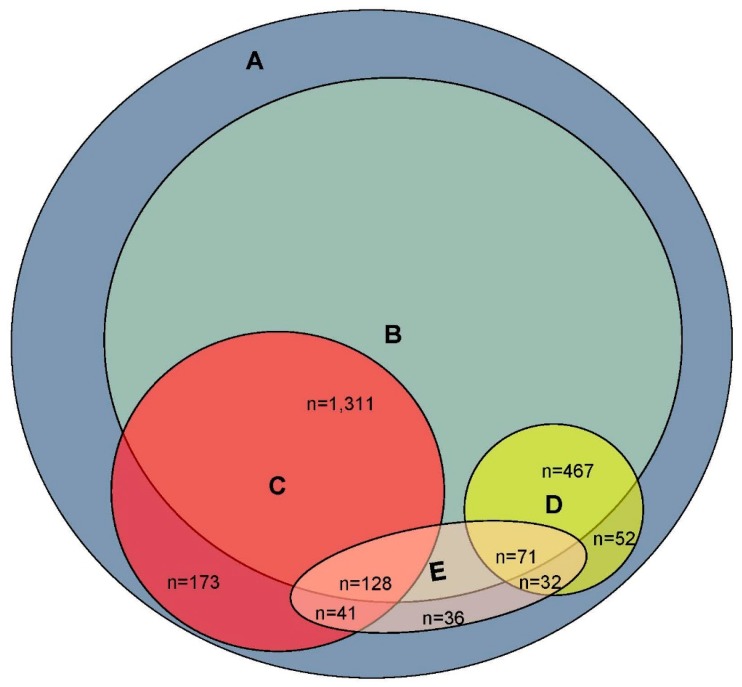
Visual representation of survey respondents classified as (Circle A) Total Valid Reports (*N* = 5769), (Circle B) No Neurologic Disorders (*N* = 4237), (Circle C) Suspected Mild Cognitive Impairment (*N* = 1653), (Circle D) Suspected Dementia (*N* = 622), and (Ellipse E) Report of Severe Memory Problems (*N* = 522). Size of each figure and area of overlap is approximately proportionate to set size.

Participants were categorized as “suspected MCI” or “suspected dementia” based on their score on the RMT. A cutting score of <0.29 was previously identified as separating persons with dementia from those without with reasonable sensitivity and specificity (76% and 74%, respectively) [17]. Therefore, the “suspected dementia” group was defined as those scoring below 0.29 on the RMT. A score between 0.30 and 0.58 was used to identify those with suspected MCI, based on data from our previous clinical validation study [17]. As can be seen in Figure 1, the DRA identified 622 persons as having suspected dementia and 1653 as having suspected MCI.

Of the 5769 valid responses, older-adult respondents were further reduced to 4237 by eliminating all those who reported a history of traumatic brain injury (with loss of consciousness >5 min.), stroke, epilepsy, Parkinson’s disease, brain tumor, or “other brain disorder”; the number with suspected dementia was reduced to 538; and the number with suspected MCI was reduced to 1439. If the respondent’s subjective complaint of impaired memory is desired as a criterion, we can further limit the population to those 522 respondents (9%) who answered “yes” to the question “do you have severe memory problems?” This would result in 71 persons with suspected dementia and 128 with suspected MCI.

## 4. Discussion

In late 2016, approximately half of the world’s population had access to the Internet [18]. This makes it a potentially useful vehicle for the initial ascertainment of persons with mild cognitive impairment or early AD who would then be screened in person for eligibility for clinical trials. We have shown here that—depending on the exclusiveness of the criteria used—our online Dementia Risk Assessment can identify between 71 and 622 persons with suspected dementia and between 128 and 1653 persons with suspected MCI from an anonymous general population sample accumulated over 22 months. A clinical trial that sought to use the DRA for first-stage screening could explicitly direct interested parties to a protected website hosting the instrument and potentially recruit many more participants.

There are, of course, significant limitations to relying on the DRA for subject recruitment and screening. First, the instrument relies on self-reported medical history and neuropsychological symptoms. In this respect, however, it does not really differ from in-person screening. Second, the RMT’s ability to distinguish persons diagnosed as having MCI from those who are cognitively normal is good (sensitivity = 80%, specificity = 75%) [17], but probably not high enough to serve as the sole cognitive test for eligibility. Third, any trial that seeks patients with more than mild dementia would probably not be able to use the DRA self-report version, as the ability to navigate to and around the web portal and respond to the DRA items appropriately would be beyond the patients’ abilities. For them, the proxy-report version [15] might be useful. These limitations notwithstanding, the online Dementia Risk Assessment may be a useful first step in patient recruitment for clinical trials of anti-dementia treatments.
